# PUBLIC HEALTH AND SOCIAL SERVICES SYSTEM: THE DISENCHANTMENT OF ISOLATED SENIOR CAREGIVERS

**DOI:** 10.1093/geroni/igz038.1420

**Published:** 2019-11-08

**Authors:** Melanie Couture, Pam Orzeck, Apostolia Petropoulos

**Affiliations:** 1 Centre for research and expertise in social gerontology, CIUSSS West-Central Montreal, Montreal, Quebec, Canada; 2 McGill University, Montreal, Quebec, Canada; 3 Jewish Eldercare Centre, Montreal, Quebec, Canada

## Abstract

Social isolation is one of the negative consequences associated with caregiving and is experienced by approximately 20% of Canadian family caregivers. Being in a public health and social services system, Canadian caregivers should normally turn to their local community service centres (CLSC) to access formal services and feel less isolated. However, studies have shown that satisfaction is low regarding accessibility and continuity of formal support services. In an effort to develop interventions that meet the needs of isolated senior caregivers, the purpose of this exploratory descriptive qualitative study was to identify challenges encountered in accessing and utilizing formal supports within the public health and social services system in Canada. Nineteen isolated senior caregivers participated in seven focus groups. Data analysis was performed using the Miles, Huberman, and Saldana (2014) approach. Results showed that isolated caregivers do not know where to get information about existing services within the formal system. Once services are found, waiting lists are linked to unbearable delays. Some caregivers are actually redirected to private services, if they can afford it. Isolated caregivers also criticize the unpredictability of the system as they face relentless changes of care providers, inadequate services and sometimes unwarranted cancellations or terminations. In addition, they find formal services lacking human sensitivity. Many of them come to the conclusion that formal services are not worthwhile and exclude themselves from the formal system. This research demonstrated that the health and social services system can actually contribute to the social isolation of senior caregivers longing for support.

